# The second national tuberculosis prevalence survey in Vietnam

**DOI:** 10.1371/journal.pone.0232142

**Published:** 2020-04-23

**Authors:** Hai Viet Nguyen, Edine W. Tiemersma, Hoa Binh Nguyen, Frank G. J. Cobelens, Alyssa Finlay, Philippe Glaziou, Cu Huy Dao, Veriko Mirtskhulava, Hung Van Nguyen, Huyen T. T. Pham, Ngoc T. T. Khieu, Petra de Haas, Nam Hoang Do, Phan Do Nguyen, Cong Van Cung, Nhung Viet Nguyen

**Affiliations:** 1 National Tuberculosis Programme, Hanoi, Vietnam; 2 Department of Global Health and Amsterdam Institute of Global Health and Development, Amsterdam University Medical Centers, Amsterdam, the Netherlands; 3 KNCV Tuberculosis Foundation, The Hague, the Netherlands; 4 Centers for Disease Control Vietnam Office, Hanoi, Vietnam; 5 Global Tuberculosis Programme, World Health Organization, Geneva, Switzerland; McGill University, CANADA

## Abstract

**Introduction:**

Tuberculosis (TB) remains a significant cause of morbidity and mortality in Vietnam. The current TB burden is unknown as not all individuals with TB are diagnosed, recorded and notified. The second national TB prevalence survey was conducted in 2017–2018 to assess the current burden of TB disease in the country.

**Method:**

Eighty-two clusters were selected using a multistage cluster sampling design. Adult (≥15 years of age) residents having lived for 2 weeks or more in the households of the selected clusters were invited to participate in the survey. The survey participants were screened for TB by a questionnaire and digital chest X-ray after providing written informed consent. Individuals with a positive symptom screen and/or chest X-ray suggestive of TB were asked to provide sputum samples to test for *Mycobacterium tuberculosis* by Ziehl-Neelsen direct light microscopy, Xpert MTB/RIF G4, BACTEC MGIT960 liquid culture and Löwenstein-Jensen solid culture. Bacteriologically confirmed TB cases were defined by an expert panel following a standard decision tree.

**Result:**

Of 87,207 eligible residents, 61,763 (70.8%) participated, and 4,738 (7.7%) screened positive for TB. Among these, 221 participants were defined as bacteriologically confirmed TB cases. The estimated prevalence of bacteriologically confirmed adult pulmonary TB was 322 (95% CI: 260–399) per 100,000, and the male-to-female ratio was 4.0 (2.8–5.8, p<0.001). In-depth interviews with the participants with TB disease showed that only 57.9% (95% CI: 51.3–64.3%) reported cough for 2 weeks or more and 32.1% (26.3–38.6%) did not report any symptom consistent with TB, while their chest X-ray results showed that 97.7% (95% CI: 94.6–99.1) had abnormal chest X-ray images suggesting TB.

**Conclusion:**

With highly sensitive diagnostics applied, this survey showed that the TB burden in Vietnam remains high. Half of the TB cases were not picked up by general symptom-based screening and were identified by chest X-ray only. Our results indicate that improving TB diagnostic capacity and access to care, along with reducing TB stigma, need to be top priorities for TB control and elimination in Vietnam.

## Introduction

Tuberculosis (TB) remains a significant cause of morbidity and mortality worldwide. According to World Health Organisation (WHO) estimates, in 2017, TB disease affected about 10 million people and claimed nearly 1.3 million lives globally [1]. However, more than a third of the estimated TB cases are missed from notification and drive the TB epidemic in the world by substantially contributing to TB transmission in the communities [1]. Better estimates of the burden of TB at the country level are needed to develop and implement evidence-based policies in a country, which ultimately follow the WHO End TB Strategy to reduce TB mortality and incidence by 95% and 90% compared to that of 2015, respectively [2]. In Vietnam, TB surveillance is done through a web-based system that was implemented since 2009 and used by TB staff nationwide from district to national level since 2015 [3]. However, a notable number of TB patients remain undiagnosed and not notified, posing a huge threat of disease transmission [4]. Therefore, national TB prevalence surveys are needed as they can inform a better estimation of TB prevalence than notification data [5]. Vietnam conducted the first national TB prevalence survey in 2006–2007 to evaluate the TB situation in the country. This survey, using conventional screening methods and diagnostics to detect TB cases, showed a prevalence of bacteriologically confirmed pulmonary TB in adults of 307 per 100,000 population [4]. The Vietnam National TB Programme (NTP) has introduced a wide range of interventions to reduce the burden of TB, including household contact investigation, TB preventive treatment, new TB drugs and diagnostics, and active case finding along with strengthening routine TB care and treatment [6], [7]. In 2017, Vietnam NTP conducted the second national TB prevalence survey. This survey aimed to assess the current TB burden in the country and to guide future actions to meet the End TB targets in 2035 using state-of-the-art TB diagnostics [2].

## Materials and methods

### Study design and population

A cross-sectional survey based on multistage cluster sampling was conducted between October 2017 and February 2018. Based on the prevalence of TB in Vietnam estimated from the first national TB prevalence survey and the annual decline rate projected by WHO [8], a cluster size of 1,000, an anticipated design effect of 1.92 and an expected participation rate of 80% with 25% relative precision, a sample size of 82,000 (i.e., 82 clusters) was calculated [9].

Districts and communes were sampled proportional to population size (PPS) based on the Vietnam 2009 national census [10], then sub-communes in the chosen communes were selected randomly. Our survey population consisted of adult residents over 15 years of age who had lived and slept in the households of selected clusters for at least 2 weeks prior to the screening day at that site.

### Screening and diagnostic procedures

A 7-day field operation was organised in each cluster starting with a one- or two-day activity to enumerate all those living in the selected sub-commune(s). During this door-to-door census, all eligible adults received an invitation card with their unique personal identification number (PIN). Invited participants were screened for pulmonary TB by chest radiograph (X-ray) and a short questionnaire asking whether they had cough or a history of TB treatment. Participants screened positive either by interview or chest X-ray (definition in the subsequent section) were asked to engage in an in-depth interview and provide two sputum samples in Falcon tubes. The first spot sample (S1) was examined with Xpert MTB/RIF (Xpert; G4 cartridge) in nearby district laboratories or directly at the survey field-site. If the Xpert result was positive for Mycobacterium tuberculosis (MTB), the participant was asked to provide an additional spot sample at handing in the morning sample, for confirmation. This was also applied in case the Xpert result was unsuccessful and there was not enough S1 sample left for re-testing. The second sample (morning–M) was kept in portable refrigerators at 2–8°C (monitored by external thermometer) and on the day of collection delivered to the national or regional reference laboratory for direct sputum smear microscopy (smear), culture using liquid media (BACTEC MGIT 960) and species identification by TBC-ID identification test (Becton Dickinson, New Jersey, USA), as well as Löwenstein-Jensen solid culture. The final culture result was mostly the MGIT result, and the Löwenstein-Jensen result was only taken into account when the MGIT result was contaminated or unavailable. Digital chest X-ray images were stored as Digital Imaging and Communications in Medicine format (DICOM) files and transferred to the Vietnam National Lung Hospital for re-reading of all X-ray images scored as abnormal by the field radiologist and 10% of those scored as normal, selected by simple random sampling in each cluster. We did not assess the prevalence of HIV infection in this survey to preserve the willingness to participate of the eligible individuals and the simplicity of our survey procedures.

### Definitions

A participant was considered to have attended the survey if he or she was an eligible resident who went through at least one of the TB screening procedures. A participant was classified as “screened-positive” and thus eligible for sputum examination if the participant had at least one of the following: cough for two weeks or more (or cough of any duration for pregnant women); self-reported TB treatment in the two years preceding the survey; and chest X-ray with abnormalities consistent with TB. A TB case was defined as a case of bacteriologically confirmed TB, classified by a panel of experts from the Vietnam NTP (including radiologists, pulmonologists and microbiologists), US Centers for Disease Control and Prevention (Vietnam Office) and KNCV Tuberculosis Foundation, based on laboratory results, chest X-ray images, TB suggestive symptoms and TB treatment history of each participant. Detailed classification of bacteriologically confirmed TB cases is presented in Table 1. A smear-positive TB case and an Xpert-positive TB case were defined as a bacteriologically confirmed TB case with a positive smear result and at least one positive Xpert result, respectively.

**Table 1:** Classification of bacteriologically confirmed cases according to expert panel decision.

Xpert result ^a^	Culture result ^b^	Chest X-ray ^c^	TB treatment history ^d^	Number of cases
MTB detected	MTB Growth	Any	Any	130
MTB not detected	MTB Growth	Abnormal	Any	48
MTB detected	No growth	Abnormal	No history	32
MTB detected	No growth	Abnormal	< 2 years	5 ^e^
MTB detected	No growth	Abnormal	> 2 years	2
MTB detected	Contaminated/missing/NTM	Abnormal	No history	4
MTB detected	Contaminated/missing/NTM	Abnormal	< 2 years	0
MTB detected	Contaminated/missing/NTM	Abnormal	> 2 years	0
**Total number**				**221**

MTB: *Mycobacterium tuberculosis*; NTM: Nontuberculous mycobacteria

^a^ Combined result of two tests if the first test failed. Does include participants with one (n = 38) and two (n = 118) Xpert results of MTB detected;

^b^ Final culture result, which is the MGIT result unless the MGIT culture was contaminated and the LJ culture result was either no growth or MTB+, then the LJ result was taken (n = 86);

^c^ Final expert panel conclusion, abnormal meaning chest X-ray with any abnormality suggesting active TB disease;

^d^ Treatment history as reported by the participant in the in-depth interview; ^d^ Two of these patients were on TB treatment at the time of the survey.

### Data collection and analysis

Participant data were directly entered through laptops and tablets at each field station and laboratory using a locally developed, bespoke web-based data entry system (Department of Biostatistics and Medical Informatics—Hanoi Medical University). All survey team members received pre-survey training on data entry, along with other procedures of fieldwork. During the field operation, data collected was uploaded directly to the main server, and reviewed daily by the data managers of each survey team and research coordinators at the central level. Digital X-ray images were transferred to the central level after the end of field operations in two consecutive clusters.

Data analysis was conducted only for the participants that were enumerated in the door-to-door census. We noticed a number of participants were added to the TB screening procedures who were not in our sampling frame, whom we excluded them from our analysis to mitigate selection bias. Survey data were analysed using Stata14 (Stata Corporation, College Station, TX, USA). The main outcome of the survey was the estimated prevalence of bacteriologically confirmed pulmonary TB among adults in Vietnam and stratified by sex, age groups, areas and regions. Analyses included multiple imputation by chained equation of missing bacteriologically confirmed TB status using a combination of predictors consisting of sex, field and central re-reading chest X-ray results, geographic area and TB treatment history to derive individual-level missing values. This way, 20 datasets were created and the average number of cases in the resulting datasets was used for calculation of the point estimates and confidence intervals based on robust standard errors. Inverse probability weighting was applied to adjust for differences in participation rate by age, sex and cluster as per recommended methods [5]. Also, post-stratification weighting was applied using the population of Vietnam in 2017 as estimated by the General Statistics Office [11] to account for the relative contribution of each participant in the survey, in order to maximize the representativeness of our study sample [12]. Based on the data derived from all three adjustment methods, adjusted prevalence rates of smear-positive TB, Xpert-positive TB and bacteriologically confirmed TB were calculated, herein further referred to as “estimated prevalence”.

Descriptive analysis for summarizing the characteristics of survey participants was performed using Stata *svy* commands to derive adjusted proportions based on inverse probability weighting and post-stratification. Statistical differences of characteristics between groups were assessed by Chi-square test and logistic regression, trends were tested for significance using the rank-sum test. We calculated point estimates and 95% confidence intervals of TB prevalence using the Stata *mim* and *svy* commands (with *pweights* specified) to correct for design-effect [13]. Confidence intervals and p-values were derived using Rubin’s rules [14]. The Wald test was used for significance testing of prevalence trends [15].

### Ethics statement

This study has been given scientific and ethical approval by the Institutional Review Board of the Vietnam National Lung Hospital, under approval letter number 62/17/CTHĐKH-ĐĐ. All invited participants were informed about the risks and benefits of the study and signed an individual written informed consent. Those who were unable to sign were asked to provide their thumbprint. All participants with at least one positive bacteriological result for TB in the survey were referred to their local district TB unit to receive treatment. They were followed up later to ensure that adequate healthcare and treatment has been received.

## Results

**Fig 1:**
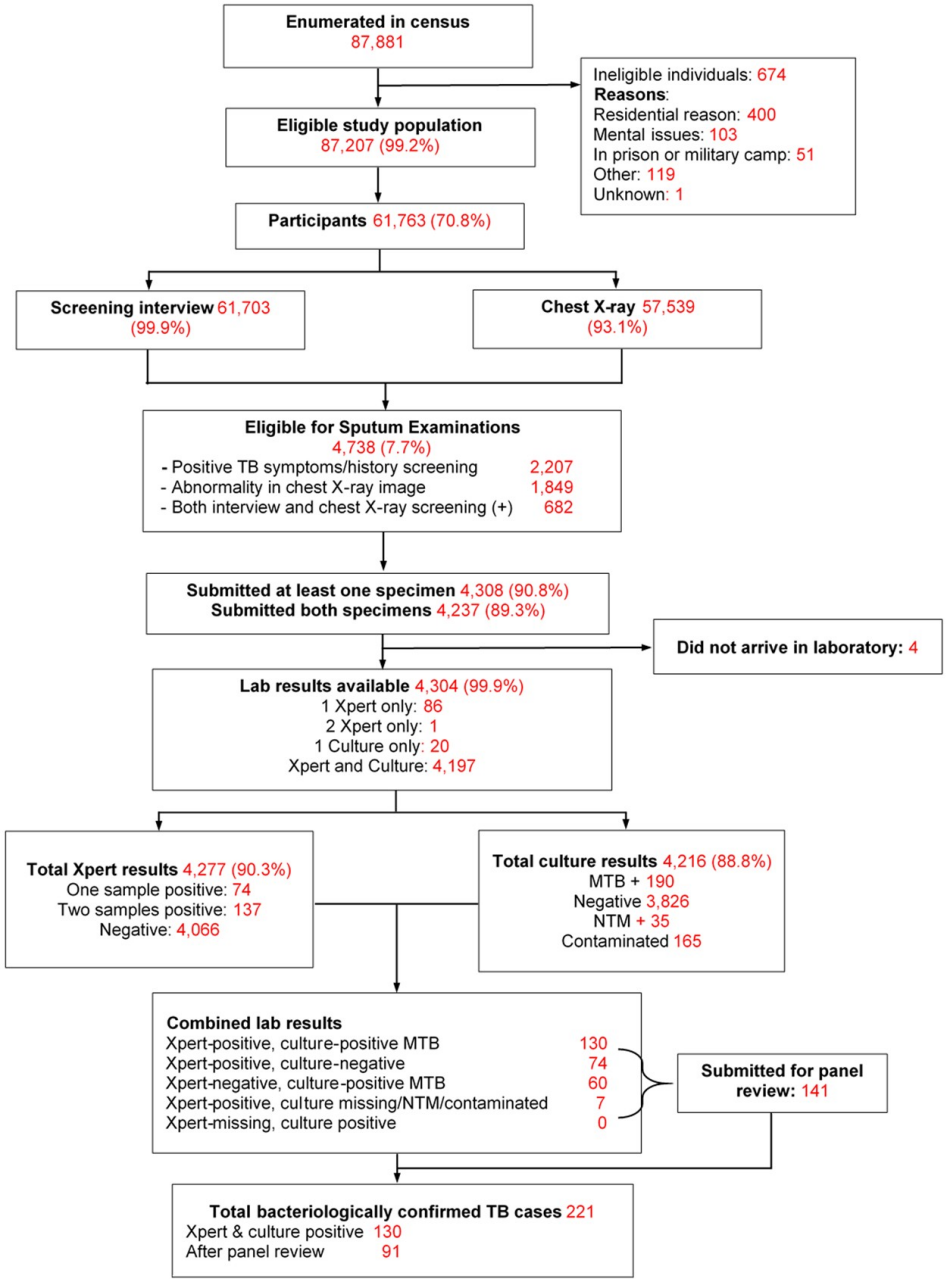
Flow diagram of the 2nd tuberculosis prevalence survey in Vietnam, 2017–2018.

**Fig 2:**
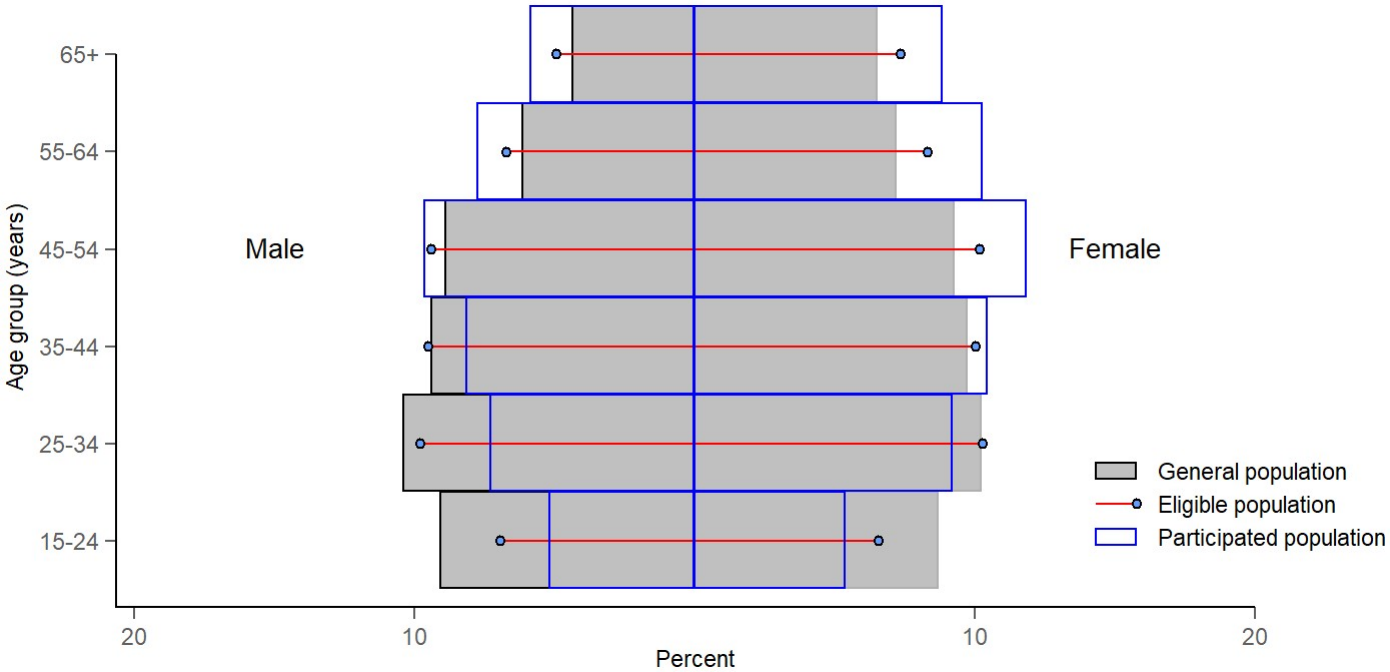
Comparison of age-sex distribution for the general, eligible and participated population.

During the census, 87,881 adult residents pre-listed on population lists obtained from the local authorities were enumerated in 82 clusters across the country. Of these, 87,207 (99.2%) were eligible to participate in the survey, of whom 61,763 (70.8%) actually participated (Fig 1). The proportions of male and female participants were 44.0% and 56.0%, respectively (Fig 2). The participation rate was higher in rural areas, in the North of Vietnam, among the elderly (age ≥ 55 years) and women (Fig 2).

Of 61,763 participants, 4,738 (7.7%) screened positive, of whom 2,118 (44.7%) only had cough ≥2 weeks, 69 (1.5%) only reported TB treatment within the preceding 2 years, 20 (0.4%) reported to have both cough ≥ 2 weeks and a history of TB treatment, and 1,849 (39.0%) were identified only by abnormal chest X-ray. The remaining 682 (14.4%) were eligible for sputum examination by both interview and chest X-ray screening (Fig 1; Table 2).

**Table 2:** Screening outcomes of the survey, by sex, age, area and region.

	Total screened participants	Total screening positive			Interview ^a^ screening positive only			Chest X-ray screening positive only			Both interview and chest X-ray positive		
		No.	%	p-value	No.	%	p-value	No.	%	p-value	No.	%	p-value
**Total**	**61,163**	**4,738**	**7.7**		**2,207**	**3.6**		**1,849**	**3.0**		**682**	**1.1**	
**Sex**				<0.001			<0.001			<0.001			<0.001
Male	27,150	2,842	10.5		1,272	4.7		1,088	4.0		482	1.8	
Female	34,613	1,896	5.5		935	2.7		761	2.2		200	0.6	
**Age group**				**<0.001**^b^			**<0.001**^b^			**<0.001**^b^			**<0.001**^b^
15–24	6,542	135	2.1	<0.001^c^	99	1.5	<0.001^c^	27	0.4	<0.001^c^	9	0.1	<0.001^c^
25–34	10,191	379	3.7	<0.001^c^	253	2.5	<0.001^c^	84	0.8	<0.001^c^	42	0.4	<0.001^c^
35–44	11,508	577	5.0	<0.001^c^	360	3.1	0.004^c^	160	1.4	<0.001^c^	57	0.5	<0.001^c^
45–54	13,289	1,074	8.1	0.045^c^	565	4.3	<0.001^c^	370	2.8	0.110^c^	139	1.1	0.468^c^
55–64	11,143	1,193	10.7	<0.001^c^	529	4.8	<0.001^c^	494	4.4	<0.001^c^	170	1.5	<0.001^c^
≥ 65	9,090	1,380	15.2	<0.001^c^	401	4.4	<0.001^c^	714	7.9	<0.001^c^	265	2.9	<0.001^c^
**Area**				**0.383**			**<0.001**			**<0.001**			**<0.001**
Urban	18,656	1,415	7.6	0.595^c^	487	2.6	<0.001^c^	710	3.8	<0.001^c^	218	1.2	0.314^c^
Rural	15,882	1,216	7.7	0.935^c^	692	4.4	<0.001^c^	403	2.5	<0.001^c^	121	0.8	<0.001^c^
Remote	27,225	2,107	7.7	0.537^c^	1,028	3.8	0.016^c^	736	2.7	<0.001^c^	343	1.3	0.001^c^
**Region**				**<0.001**			**<0.001**			**<0.001**			**<0.001**
North	25,575	1,917	7.5	<0.001^c^	1,094	4.3	<0.001^c^	639	2.5	<0.001^c^	184	0.7	<0.001^c^
Central	13,525	1,216	9.0	0.168^c^	531	3.9	<0.001^c^	391	2.9	0.428^c^	294	2.2	<0.001^c^
South	22,663	1,605	7.1	<0.001^c^	582	2.6	0.012^c^	819	3.6	<0.001^c^	204	0.9	<0.001^c^

No: number of participants with such characteristics

^a^ Including those who reported TB symptoms and/or TB treatment history, defined as follows:
*TB symptoms*: cough for 2 weeks or more, or cough of any duration in pregnant women. *Treatment history*: currently on TB treatment or reporting TB treatment in the 2 years preceding the survey

^b^ p-value for trend, obtained by rank-sum test

^c^ p-value for the difference between this age group/ area/ region with other groups combined, obtained by Pearson’s chi-squared test

Of the 4,738 participants who screened-positive, 4,304 (90.8%) had at least one sputum sample tested with Xpert or MGIT culture (Fig 1). Of 4,277 (90.3%) participants who had at least one Xpert test result, 74 (1.7%) had one sample positive for MTB, 137 (3.2%) had two samples positive for MTB, and 4,066 had only negative Xpert test results. Culture results were available for 4,216 (88.8%) participants, of which 190 (4.5%) showed MTB growth. There were also 4,216 smear results, of which 77 (1.8%) were positive for acid-fast bacilli. Overall, 130 participants had positive results for MTB on both Xpert and MGIT culture. An additional 91 participants with discordant laboratory results (Xpert-positive with culture-negative or vice versa) were identified as TB cases by the expert panel, bringing the total number of bacteriologically confirmed survey TB cases to 221 (Fig 1; Table 1). Among these 221 TB cases, 5 (2.3%) were on TB treatment at the time of the survey (all Xpert(+)/culture(+)); and 7 (3.1%) had a history of TB treatment within two years preceding the survey, with 2 cases Xpert(+)/culture(-) and 5 cases Xpert(+)/culture(+). There were also 19 cases (8.6%) reported to have a history of TB treatment that started more than two years preceding the survey, bringing the total of previously treated TB cases to 31 (14.0%, Table 1).

In-depth interviews were performed for the participants who screened positive, including all 221 bacteriologically confirmed cases. Of these 221, in the in-depth interview, 148 (67.0%, CI: 60.5–72.9) reported cough of any duration, 20 (9.1%, CI: 5.9–13.7) reported cough of less than 2 weeks while 128 (57.9%, CI: 51.3–64.3) reported cough of 2 weeks or more and 135 (61.1%, CI: 54.4–67.3) reported productive cough with sputum. Aside from coughing, 43 (19.5%, CI: 14.7–25.3) TB cases reported other TB symptoms, including haemoptysis (7 cases), weight loss (17 cases), fever (28 cases) and night sweats (17 cases). Among the 221 cases, 71 (32.1%, CI: 26.3–38.6) did not report any symptom suggestive of TB (Table 3).

**Table 3:** TB symptoms reported by bacteriologically confirmed TB cases in the in-depth interview.

TB symptoms	TB patients reported having such symptoms	Proportion (%)	95% confidence interval
Cough (any duration)	148	67.0	60.5–72.9
Cough < 2 weeks	20	9.1	5.9–13.7
Cough ≥ 2 weeks	128	57.9	51.3–64.3
Cough with sputum	135	61.1	54.4–67.3
Haemoptysis	7	3.2	1.5–6.5
Fever	28	12.7	8.9–17.8
Weight loss	17	7.7	4.8–12.1
Night sweats	17	7.7	4.8–12.1
Any symptoms	150	67.9	61.4–73.7
No symptoms reported	71	32.1	26.3–38.6
**Total TB cases**	**221**^a^		

^a^ Number of TB cases found in the survey. As one TB case could have more than one symptom, the numbers do not add up to 221.

Missing data were imputed for bacteriologically confirmed TB status. There were 430 screened positive participants who did not hand in any sputum sample tested during the survey and 4 participants whose laboratory results were not available, resulting in 434 records (9.2%) that required imputation. After imputation and adjustment by inverse probability weighting and post-stratification, the estimated prevalence of smear-positive TB was 79 per 100,000 adults (95% CI: 55–115; Table 4). The estimated adjusted prevalence of Xpert-positive TB was 235 (187–296) per 100,000 adults, and that of bacteriologically confirmed TB was 322 (260–399) per 100,000 adults (Table 4). The effect of this adjustment was a 15–16% lower prevalence compared to the crude estimates (Table 5).

**Table 4:** Estimated prevalence of pulmonary tuberculosis in Vietnam, overall and by sex, age, area and region.

		Smear-positive TB				Xpert-positive TB				Bacteriologically confirmed TB			
	Number of participants	No. of cases	Crude prevalence (per 100,000) n (95% CI)	Weighted prevalence (per 100,000)^a^ n (95% CI)	p-value	No. of cases	Crude prevalence (per 100,000) n (95% CI)	Weighted prevalence (per 100,000)^a^ n (95% CI)	p-value	No. of cases	Crude prevalence (per 100,000) n (95% CI)	Weighted prevalence (per 100,000)^a^ n (95% CI)	p-value
**Total**	**61**,**763**	**50**	**81 (61–107)**	**79 (55–115)**		**173**	**280 (241–325)**	**235 (187–296)**		**221**	**358 (314–408)**	**322 (260–399)**	
**Sex**					0.004				**<0.001**				<0.001
Male	27,150	34	125 (89–175)	118 (78–180)		138	508 (430–600)	390 (309–491)		175	645 (556–747)	522 (420–648)	
Female	34,613	16	46 (28–75)	42 (23–79)		35	101 (72–141)	89 (60–134)		46	133 (100–177)	133 (89–198)	
**Age group**					**<0.001**^b^				**<0.001**^b^				**0.001**^b^
15–24	6,542	0	0	0		4	61 (23–163)	49 (19–127)	<0.001^c^	4	61 (23–163)	63 (18–226)	0.007^c^
25–34	10,191	9	88 (46–170)	109 (55–218)	0.218^c^	13	128 (74–220)	142 (76–264)	0.051^c^	17	167 (104–268)	203 (114–360)	0.049^c^
35–44	11,508	8	70 (35–139)	69 (35–134)	0.637^c^	21	182 (119–280)	185 (111–308)	0.263^c^	31	269 (190–383)	287 (180–454)	0.556^c^
45–54	13,289	15	113 (68–187)	123 (72–211)	0.056^c^	42	316 (233–427)	344 (239–496)	0.034^c^	50	376 (285–496)	423 (300–597)	0.113^c^
55–64	11,143	13	117 (68–201)	130 (68–251)	0.116^c^	52	466 (356–612)	505 (365–698)	<0.001^c^	62	556 (434–713)	618 (463–823)	<0.001^c^
≥ 65	9,090	5	55 (23–132)	55 (18–167)	0.515^c^	41	451 (332–612)	446 (317–629)	<0.001^c^	57	627 (484–812)	689 (511–930)	<0.001^c^
**Stratum**					**0.034**				**0.004**				**0.024**
Urban	18,656	27	145 (99–211)	140 (86–228)	0.008^c^	75	402 (321–504)	342 (252–463)	0.008^c^	90	482 (393–593)	445 (327–604)	0.020^c^
Rural	15,882	13	82 (48–141)	76 (44–133)	0.893^c^	44	277 (206–372)	247 (148–414)	0.818^c^	52	327 (250–429)	309 (181–526)	0.852^c^
Remote	27,225	10	37 (20–68)	37 (15–93)	0.032^c^	54	198 (152–259)	152 (108–215)	0.003^c^	79	290 (233–362)	241 (182–572)	0.027^c^
**Region**					**0.129**				**0.159**				**0.297**
North	25,575	10	39 (21–73)	44 (22–86)	0.131^c^	49	192 (145–253)	168 (105–267)	0.102^c^	70	274 (217–346)	262 (180–380)	0.173^c^
Central	13,525	14	104 (61–175)	92 (48–176)	0.044^c^	42	311 (230–420)	253 (152–423)	0.067^c^	49	362 (274–479)	316 (181–552)	0.939^c^
South	22,663	26	115 (78–168)	106 (62–182)	0.640^c^	82	362 (292–449)	289 (216–385)	0.749^c^	102	450 (371–546)	380 (287–504)	0.163^c^

No: number CI: confidence interval N/A: Not available

^a^ Weighted prevalence: estimated prevalence after missing data imputation, inverse probability weighting and post-stratification (see more in the *Data collection and analysis* section)

^b^ p-value for trend, obtain by Wald test

^c^ p-value for the difference between this age group/ area/ region with other groups combined, obtained with logit regression

**Table 5:** Comparing the prevalence of bacteriologically confirmed TB with and without missing data imputation, inverse probability weighting and post-stratification.

	Prevalence of bacteriologically confirmed TB without missing data imputation (per 100,000)	Prevalence of bacteriologically confirmed TB with missing data imputation (per 100,000)
**No weight**	358 (95% CI: 295–434)	380 (95% CI: 315–458)
**Inverse probability weighting (IPW)**	341 (95% CI: 279–417)	365 (95% CI: 301–444)
**IPW & post-stratification**	299 (95% CI: 242–370)	322 (95% CI: 260–398)

CI: confidence interval IPW: Inverse probability weighting

**Fig 3:**
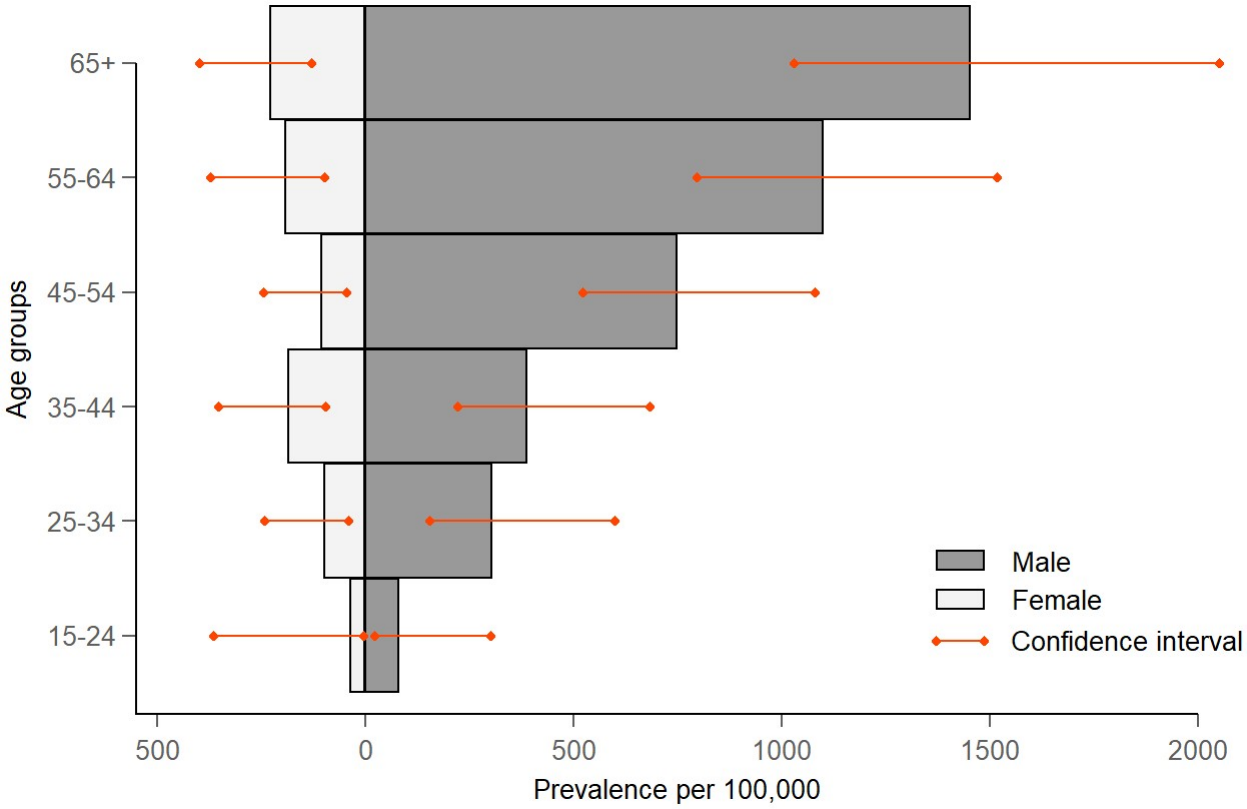
Comparison of the prevalence of bacteriologically confirmed tuberculosis per 100,000 population between males and females by age group.

Subgroup analysis by sex and age showed a significant, large difference in the prevalence of TB between men and women (Fig 3). Overall, the male-to-female ratio for the prevalence of smear-positive TB was 2.8 (1.4–5.6), while this ratio was 4.4 (3.0–6.4) for Xpert-positive TB and 4.0 (2.8–5.8) for bacteriologically confirmed TB (Table 6). There was a significantly increasing trend in the prevalence of TB with increasing age (p<0.001, Table 4). Also, the estimated prevalence of TB was significantly higher in urban areas compared to rural and remote areas (p = 0.020), and lower in remote areas than in urban and rural areas (p = 0.027, Table 4). The estimated prevalence of TB was highest in the southern region, although no statistically significant difference between regions was observed (Table 4).

**Table 6:** Odds ratio of TB for men compared to women by age group.

Age group	Xpert MTB/Rif positive		Sputum smear-positive		Bacteriologically confirmed	
	OR (95% CI)	p-value	OR (95% CI)	p-value	OR (95% CI)	p-value
**Total**	**4.4 (3.0–6.4)**	**<0.001**	**2.8 (1.4–5.6)**	**0.004**	**4.0 (2.8–5.8)**	**<0.001**
15–24	3.2 (0.3–32.9)	0.324	N/A		2.2 (0.2–32.4)	0.554
25–34	3.1 (0.8–11.8)	0.090	3.9 (0.7–20.7)	0.104	3.2 (1.0–9.7)	0.041
35–44	2.3 (0.8–6.5)	0.114	1.7 (0.4–7.6)	0.446	2.1 (0.9–4.9)	0.074
45–54	6.8 (2.4–19.0)	<0.001	2.9 (0.8–10.3)	0.101	7.1 (2.9–17.5)	<0.001
55–64	6.4 (2.8–14.8)	<0.001	2.4 (0.7–8.2)	0.178	5.7 (2.7–12.2)	<0.001
≥ 65	6.4 (3.0–14.1)	<0.001	4.2 (0.8–21.6)	0.085	6.5 (3.4–12.5)	<0.001

N/A: Not available OR: Odds ratio of TB for men compared to women

## Discussion

The overall estimated prevalence of bacteriologically confirmed TB among adults in Vietnam was 322 per 100,000 population. The prevalence of bacteriologically confirmed TB was 4 times higher in males compared to females and increased with age. Urban areas and the southern region of Vietnam had the highest prevalence of TB. The findings in this survey were consistent with the results of the TB prevalence survey in Vietnam done in 2007 in terms of the age, sex and regional trends of TB [4]. The present survey showed a lower prevalence of TB compared to several other surveys done in high TB-burden countries in Asia over the last 5 years, for example, Indonesia (759/100,000 adults, CI: 590–961) in 2014 [16], Philippines (1,159/100,000 adults, CI: 1,016–1,301) in 2016 [17], and Myanmar (468/100,000 adults, CI: 390–545) in 2017 [18]. However, screening methods differed across these surveys. While our survey applied Xpert MTB/Rif for all screened positive participants, Xpert Ultra was applied in the Myanmar and Philippines surveys, and in the Indonesia survey, Xpert MTB/Rif was only used to confirm the result of positive sputum smear microscopy [19]. Therefore, direct comparisons between our findings and the results of other surveys are not possible. To accurately compare the prevalence of TB found in our survey with that in others, especially the previous survey in Vietnam in 2006–2007, analyses are needed that adjust for differences in sensitivity and specificity of screening and diagnostic methods applied.

The HIV status of the participants was not assessed in our study because the invasive HIV blood test could impede the willingness to participate in the survey and further complicate our procedures. Also, self-reported HIV status is unreliable in Vietnam due to the high level of HIV-related stigma [20]. However, the lack of HIV data is unlikely to have biased our result, since the prevalence of individual with TB-HIV co-infection in Vietnam is low, only 2.7% among notified TB cases in 2017 [21].

In-depth interview data showed that only 57.9% of the survey TB cases reported having cough symptoms for two weeks or more, meaning that the remaining cases would have been missed by general symptom-based diagnostic criteria as applied currently. Most TB cases were screened positive for TB by chest X-ray, thus eligible for sputum examination with Xpert and culture. The significant proportion of abnormal chest X-ray images among bacteriologically confirmed TB cases (97.7%, S1 Table) and the relatively low cost of chest X-ray in Vietnam [22] suggest it also has an important potential role in active case finding in the country.

Results found in this study indicated that a relatively big gap of TB case detection in Vietnam should be addressed, as only 31 (14.0%) of the TB patients identified in the survey were currently on TB treatment or had been treated for TB, while the majority (86.0%) had not yet accessed any TB care and thus were unknown to the Vietnam NTP. This is in line with the result of the first survey, in which 78% of the smear-positive TB case found were not known to the NTP [4]. Similar proportions of TB patients who had not accessed were found in the Philippines (77%) [17] and Myanmar (95%) [18]. Although all TB patients found in this survey were referred to NTP healthcare facilities, it is unclear how many of them would seek TB care if they had not participated in the survey. This gap in case detection is mainly due to the insufficient awareness of TB disease and the lack of access to health care that is linked to the NTP [23]. Also, stigma is a major contributor, as it has considerable impacts including delays in seeking health care [24]. In addition, 33% of TB cases did not report any symptom suggestive of TB during the survey screening. This further illustrates the drawbacks of TB case detection based on symptoms only [25], [26].

Within the past decade, active case finding activities in Vietnam have been implemented and scaled up with promising results, including household contact investigation, TB screening for patients with HIV and non-communicable diseases, and mobile chest X-ray screening with Xpert examination (the Double X Strategy) for high-risk populations, for example, inmates in prisons, coal miners and elderly people [27], [28]. At the time of the survey, active case finding in the community using the Double X Strategy had been implemented in four cities in Vietnam, which are Hai Phong, Hoi An, Ca Mau and Ho Chi Minh City [29], [30]. Although it was only piloted on a small scale, this strategy had a promising impact on case finding. For instance, in a TB screening campaign on Cu Lao Cham Island, 17 new TB cases were found compared to 2 active cases on the island before the campaign took place [31]. This is in contrast with several studies that showed that symptom-based active case finding was not effective [32–36]. This result, along with our finding, suggests that promotion and scale-up active case finding using the Double X Strategy of the Vietnam NTP may be particularly effective in finding the missed cases.

Nearly one-fourth of the total estimated prevalence of bacteriologically confirmed TB was due to smear-positive TB. This finding is similar to that of other recent surveys in the region, for example in the Philippines (37.1%) in 2016 [17] and Myanmar (13.0%) in 2017 [18]. None of the smear-positive cases were found in the youngest age group (15–24 years of age), and also the prevalence of bacteriologically confirmed TB was lower compared to that of older age groups.

The male-to-female ratio in the prevalence of bacteriologically confirmed TB found in this survey was exceptionally high, reaching 4:1. This ratio was in line with the finding of the first TB prevalence survey of Vietnam in 2007 [4], higher than in the recent surveys in the region, e.g., in the survey in the Philippines in 2016 (2.5:1) [17], and higher than the average male-to-female ratio found in 56 previous TB prevalence surveys (2.2:1) [37]. The gender gap in TB prevalence found in this study was similar, and even higher in older age groups compared to the notification data of Vietnam in 2017 (S1 Fig), implying that the difference between sexes in Vietnam reflects a true discrepancy in disease occurrence. The reason behind this phenomenon is still controversial, as it is unknown whether because of the sociocultural and health behaviour related reasons that heighten the chance of transmitting the disease among men, or due to the differences in the biological structure of the respiratory tract and immunological responses between men and women [38]. Interestingly, the male-to-female ratio increased with age, suggesting that age is also a predictor for the TB prevalence difference between men and women. The increase in the TB prevalence male-to-female ratio along with increasing age can potentially be explained by the accumulation of the established TB risk factors over time, for example, smoking gradually damages the lungs and affects the immune response over decades, making long-time smokers more susceptible to TB disease [39].

Our study had limitations. Firstly, there was a relatively low participation rate in young age groups. This might have been caused by the duration of screening days, as screening always took place on weekdays, when eligible residents had to work, which may have hindered the participation in younger age groups. To minimise the bias caused by this, our survey teams worked extra time to perform screening procedures in the evening and encouraged younger eligible residents to participate in the survey after their daily work. Secondly, the overall participation rate was lowered by 14.0% due to the erroneous inclusion of participants who were not in the sampling frame by some teams to reach targets, thus had to be excluded from our analysis. Our survey teams obtained resident lists from local authorities before doing the door-to-door household census in the first days of field operations. As some of the resident lists were quite outdated, our survey teams had to resort to additional sampling during the census. However, we observed that there were a number of participants who may have been included during screening days to reach workload targets because they were symptomatic and willing to attend the survey to receive free health care, therefore, these participants were excluded from our analysis.

## Conclusion

The second TB prevalence survey in Vietnam showed that the burden of TB in Vietnam remains high. The high prevalence of Xpert MTB/Rif-positive pulmonary TB suggests that active case finding should be promoted and scaled up nationally, giving the missing TB patients more opportunities to be diagnosed promptly. The NTP needs to prioritize strengthening TB diagnostic capacity in all health facilities, improve access to health care for high-risk populations and reduce the stigma of TB on a national level. Our survey indicates that continued and adamant political commitment to support TB control activity technically and financially is needed to reach the targets towards TB elimination in Vietnam.

## Supporting information

S1 Table Field chest X-ray result among bacteriologically confirmed TB cases. (DOCX)

S2 Table Survey participation rate by sex, age, area and region. (DOCX)

S1 Fig Comparing the prevalence of TB found in the survey with notification data of Vietnam in 2017, by age and sex. (TIF)

S1 Text Consent form used in the survey. (DOCX)

S2 Text Screening and in-depth questionnaires used in the survey. (DOCX)
