# Correction: The second national tuberculosis prevalence survey in Vietnam

**DOI:** 10.1371/journal.pone.0236532

**Published:** 2020-07-16

**Authors:** Hai Viet Nguyen, Edine W. Tiemersma, Hoa Binh Nguyen, Frank G. J. Cobelens, Alyssa Finlay, Philippe Glaziou, Cu Huy Dao, Veriko Mirtskhulava, Hung Van Nguyen, Huyen T. T. Pham, Ngoc T. T. Khieu, Petra de Haas, Nam Hoang Do, Phan Do Nguyen, Cong Van Cung, Nhung Viet Nguyen

In Table 1, the data and footnotes use an outdated analysis and do not correctly describe the contents of the table. Please see the correct Table 1 here.

**Table 1:** Classification of bacteriologically confirmed cases according to expert panel decision.

Xpert result ^a^	Culture result ^b^	Chest X-ray ^c^	TB treatment history ^d^	Number of cases
MTB detected	MTB Growth	Any	Any	130 ^e^
MTB not detected	MTB Growth	Abnormal	Any	48
MTB detected	No growth	Abnormal	No history	34
MTB detected	No growth	Abnormal	< 2 years	2
MTB detected	No growth	Abnormal	> 2 years	3
MTB detected	Contaminated/missing/NTM	Abnormal	No history	4
MTB detected	Contaminated/missing/NTM	Abnormal	< 2 years	0
MTB detected	Contaminated/missing/NTM	Abnormal	> 2 years	0
**Total number**				**221**

MTB: *Mycobacterium tuberculosis*; NTM: Nontuberculous mycobacteria

^a^ Combined result of two Xpert tests on different sputum sample (M1 and M1b). Does include participants with one (n = 55) and two (n = 118) Xpert results of MTB detected

^b^ Final culture result, which is the MGIT result unless the MGIT culture was contaminated and the LJ culture result was either no growth or MTB+, then the LJ result was taken (n = 1)

^c^ Final expert panel conclusion, abnormal meaning chest X-ray with any abnormality suggesting active TB disease

^d^ Treatment history as reported by the participant in the in-depth interview

^e^ Five of these patients were on TB treatment at the time of the survey, and five reported to have TB treatment history within two years preceding the survey.

In Table 2 and Table 4, the headings Rural and Remote are swapped in the fifteenth and sixteenth rows. The fifteenth row should be Remote and the sixteenth row should be Rural. Please see the correct Table 2 and Table 4 here.

**Table 2:** Screening outcomes of the survey, by sex, age, area, and region.

	Total screened participants	Total screening positive			Interview ^a^ screening positive only			Chest X-ray screening positive only			Both interview and chest X-ray positive		
		No.	%	p-value	No.	%	p-value	No.	%	p-value	No.	%	p-value
**Total**	**61,163**	**4,738**	**7.7**		**2,207**	**3.6**		**1,849**	**3.0**		**682**	**1.1**	
**Sex**				<0.001			<0.001			<0.001			<0.001
Male	27,150	2,842	10.5		1,272	4.7		1,088	4.0		482	1.8	
Female	34,613	1,896	5.5		935	2.7		761	2.2		200	0.6	
**Age group**				**<0.001**^**b**^			**<0.001**^**b**^			**<0.001**^**b**^			**<0.001**^**b**^
15–24	6,542	135	2.1	<0.001^c^	99	1.5	<0.001^c^	27	0.4	<0.001^c^	9	0.1	<0.001^c^
25–34	10,191	379	3.7	<0.001^c^	253	2.5	<0.001^c^	84	0.8	<0.001^c^	42	0.4	<0.001^c^
35–44	11,508	577	5.0	<0.001^c^	360	3.1	0.004^c^	160	1.4	<0.001^c^	57	0.5	<0.001^c^
45–54	13,289	1,074	8.1	0.045^c^	565	4.3	<0.001^c^	370	2.8	0.110^c^	139	1.1	0.468^c^
55–64	11,143	1,193	10.7	<0.001^c^	529	4.8	<0.001^c^	494	4.4	<0.001^c^	170	1.5	<0.001^c^
≥ 65	9,090	1,380	15.2	<0.001^c^	401	4.4	<0.001^c^	714	7.9	<0.001^c^	265	2.9	<0.001^c^
**Area**				**0.383**			**<0.001**			**<0.001**			**<0.001**
Urban	18,656	1,415	7.6	0.595^c^	487	2.6	<0.001^c^	710	3.8	<0.001^c^	218	1.2	0.314^c^
Remote	15,882	1,216	7.7	0.935^c^	692	4.4	<0.001^c^	403	2.5	<0.001^c^	121	0.8	<0.001^c^
Rural	27,225	2,107	7.7	0.537^c^	1,028	3.8	0.016^c^	736	2.7	<0.001^c^	343	1.3	0.001^c^
**Region**				**<0.001**			**<0.001**			**<0.001**			**<0.001**
North	25,575	1,917	7.5	<0.001^c^	1,094	4.3	<0.001^c^	639	2.5	<0.001^c^	184	0.7	<0.001^c^
Central	13,525	1,216	9.0	0.168^c^	531	3.9	<0.001^c^	391	2.9	0.428^c^	294	2.2	<0.001^c^
South	22,663	1,605	7.1	<0.001^c^	582	2.6	0.012^c^	819	3.6	<0.001^c^	204	0.9	<0.001^c^

No: number of participants with such characteristics

^a^ Including those who reported TB symptoms and/or TB treatment history, defined as follows:

*TB symptoms*: cough for 2 weeks or more, or cough of any duration in pregnant women.

*Treatment history*: currently on TB treatment or reporting TB treatment in the 2 years preceding the survey

^b^ p-value for trend, obtained by rank-sum test

^c^ p-value for the difference between this age group/ area/ region with other groups combined, obtained by Pearson’s chi-squared test

**Table 4:** Estimated prevalence of pulmonary tuberculosis in Vietnam, overall and by sex, age, area and region.

		Smear-positive TB				Xpert-positive TB				Bacteriologically confirmed TB			
	Number of participants	No. of cases	Crude prevalence (per 100,000) n (95% CI)	Weighted prevalence (per 100,000)^a^ n (95% CI)	p-value	No. of cases	Crude prevalence (per 100,000) n (95% CI)	Weighted prevalence (per 100,000)^a^ n (95% CI)	p-value	No. of cases	Crude prevalence (per 100,000) n (95% CI)	Weighted prevalence (per 100,000)^a^ n (95% CI)	p-value
**Total**	**61**,**763**	**50**	**81 (61–107)**	**79 (55–115)**		**173**	**280 (241–325)**	**235 (187–296)**		**221**	**358 (314–408)**	**322 (260–399)**	
**Sex**					0.004				**<0.001**				<0.001
Male	27,150	34	125 (89–175)	118 (78–180)		138	508 (430–600)	390 (309–491)		175	645 (556–747)	522 (420–648)	
Female	34,613	16	46 (28–75)	42 (23–79)		35	101 (72–141)	89 (60–134)		46	133 (100–177)	133 (89–198)	
**Age group**					**<0.001**^**b**^				**<0.001**^**b**^				**0.001**^**b**^
15–24	6,542	0	0	0		4	61 (23–163)	49 (19–127)	<0.001^c^	4	61 (23–163)	63 (18–226)	0.007^c^
25–34	10,191	9	88 (46–170)	109 (55–218)	0.218^c^	13	128 (74–220)	142 (76–264)	0.051^c^	17	167 (104–268)	203 (114–360)	0.049^c^
35–44	11,508	8	70 (35–139)	69 (35–134)	0.637^c^	21	182 (119–280)	185 (111–308)	0.263^c^	31	269 (190–383)	287 (180–454)	0.556^c^
45–54	13,289	15	113 (68–187)	123 (72–211)	0.056^c^	42	316 (233–427)	344 (239–496)	0.034^c^	50	376 (285–496)	423 (300–597)	0.113^c^
55–64	11,143	13	117 (68–201)	130 (68–251)	0.116^c^	52	466 (356–612)	505 (365–698)	<0.001^c^	62	556 (434–713)	618 (463–823)	<0.001^c^
≥ 65	9,090	5	55 (23–132)	55 (18–167)	0.515^c^	41	451 (332–612)	446 (317–629)	<0.001^c^	57	627 (484–812)	689 (511–930)	<0.001^c^
**Area**					**0.034**				**0.004**				**0.024**
Urban	18,656	27	145 (99–211)	140 (86–228)	0.008^c^	75	402 (321–504)	342 (252–463)	0.008^c^	90	482 (393–593)	445 (327–604)	0.020^c^
Remote	15,882	13	82 (48–141)	76 (44–133)	0.893^c^	44	277 (206–372)	247 (148–414)	0.818^c^	52	327 (250–429)	309 (181–526)	0.852^c^
Rural	27,225	10	37 (20–68)	37 (15–93)	0.032^c^	54	198 (152–259)	152 (108–215)	0.003^c^	79	290 (233–362)	241 (182–572)	0.027^c^
**Region**					**0.129**				**0.159**				**0.297**
North	25,575	10	39 (21–73)	44 (22–86)	0.131^c^	49	192 (145–253)	168 (105–267)	0.102^c^	70	274 (217–346)	262 (180–380)	0.173^c^
^c^entral	13,525	14	104 (61–175)	92 (48–176)	0.044^c^	42	311 (230–420)	253 (152–423)	0.067^c^	49	362 (274–479)	316 (181–552)	0.939^c^
South	22,663	26	115 (78–168)	106 (62–182)	0.640^c^	82	362 (292–449)	289 (216–385)	0.749^c^	102	450 (371–546)	380 (287–504)	0.163^c^

No: number; CI: confidence interval; N/A: Not available

^a^ Weighted prevalence: estimated prevalence after missing data imputation, inverse probability weighting and post-stratification (see more in the *Data collection and analysis* section)

^b^ p-value for trend, obtain by Wald test

^c^ p-value for the difference between this age group/area/region with other groups combined, obtained with logit regression
