# Supplementary material for: The second national tuberculosis prevalence survey in Vietnam
Source: PLoS One. 2020 Apr 23;15(4):e0232142. doi: 10.1371/journal.pone.0232142 (PMC7179905; doi:10.1371/journal.pone.0232142)
Supplement: S1 Table — (DOCX) [file pone.0232142.s001.docx]

**S1 Table. Field chest X-ray result among bacteriologically confirmed TB cases.**

|  | **Bacteriologically confirmed TB case** | | | | |
| --- | --- | --- | --- | --- | --- |
| **Field chest X-ray result** | Yes | | No | | **Total** |
|  | n | % | n | **%** |  |
| Abnormal | 215 | 97.7 | 2,316 | 4.0 | 2,531 |
| Normal^a^ | 5 | 2.3 | 55,003 | 96.0 | 55,008 |
| **Total** | **220^b^** | **100** | **57,319** | **100** | **57,539** |

^a^ Including other abnormal chest X-ray images, not suggesting TB

^b^ One TB case did not have chest X-ray taken
