# Supplementary material for: The second national tuberculosis prevalence survey in Vietnam
Source: PLoS One. 2020 Apr 23;15(4):e0232142. doi: 10.1371/journal.pone.0232142 (PMC7179905; doi:10.1371/journal.pone.0232142)
Supplement: S2 Table — (DOCX) [file pone.0232142.s002.docx]

**S2 Table. Survey participation rate by sex, age, area and region.**

|  | **Enumerated population (n)** | **Eligible population (n)** | **Participants**  **(n)** | **Participation rate (%)** |
| --- | --- | --- | --- | --- |
| **Total** | **87,881** | **87,207** | **61,763** | **70.8** |
| **Sex** |  |  |  |  |
| Male | 41,642 | 41,288 | 34,613 | 65.8 |
| Female | 46,239 | 45,919 | 27,150 | 75.4 |
| **Age group** |  |  |  |  |
| 15-24 | 11,884 | 11,771 | 6,542 | 55.6 |
| 25-34 | 17,633 | 17,500 | 10,191 | 58.2 |
| 35-44 | 17,170 | 17,039 | 11,508 | 67.5 |
| 45-54 | 17,151 | 17,065 | 13,289 | 77.9 |
| 55-64 | 13,176 | 13,107 | 11,143 | 85.0 |
| ≥ 65 | 10,867 | 10,725 | 9,090 | 84.8 |
| **Area** |  |  |  |  |
| Urban | 28,446 | 28,262 | 18,656 | 66.0 |
| Rural | 20,700 | 20,623 | 15,882 | 77.0 |
| Remote | 38,735 | 38,322 | 27,225 | 71.0 |
| **Region** |  |  |  |  |
| North | 32,437 | 32,384 | 25,575 | 79.0 |
| Central | 17,439 | 17,421 | 13,525 | 77.6 |
| South | 38,005 | 37,402 | 22,663 | 60.6 |
