# Supplementary figures and images for: The second national tuberculosis prevalence survey in Vietnam
Source: PLoS One. 2020 Apr 23;15(4):e0232142. doi: 10.1371/journal.pone.0232142 (PMC7179905; doi:10.1371/journal.pone.0232142)

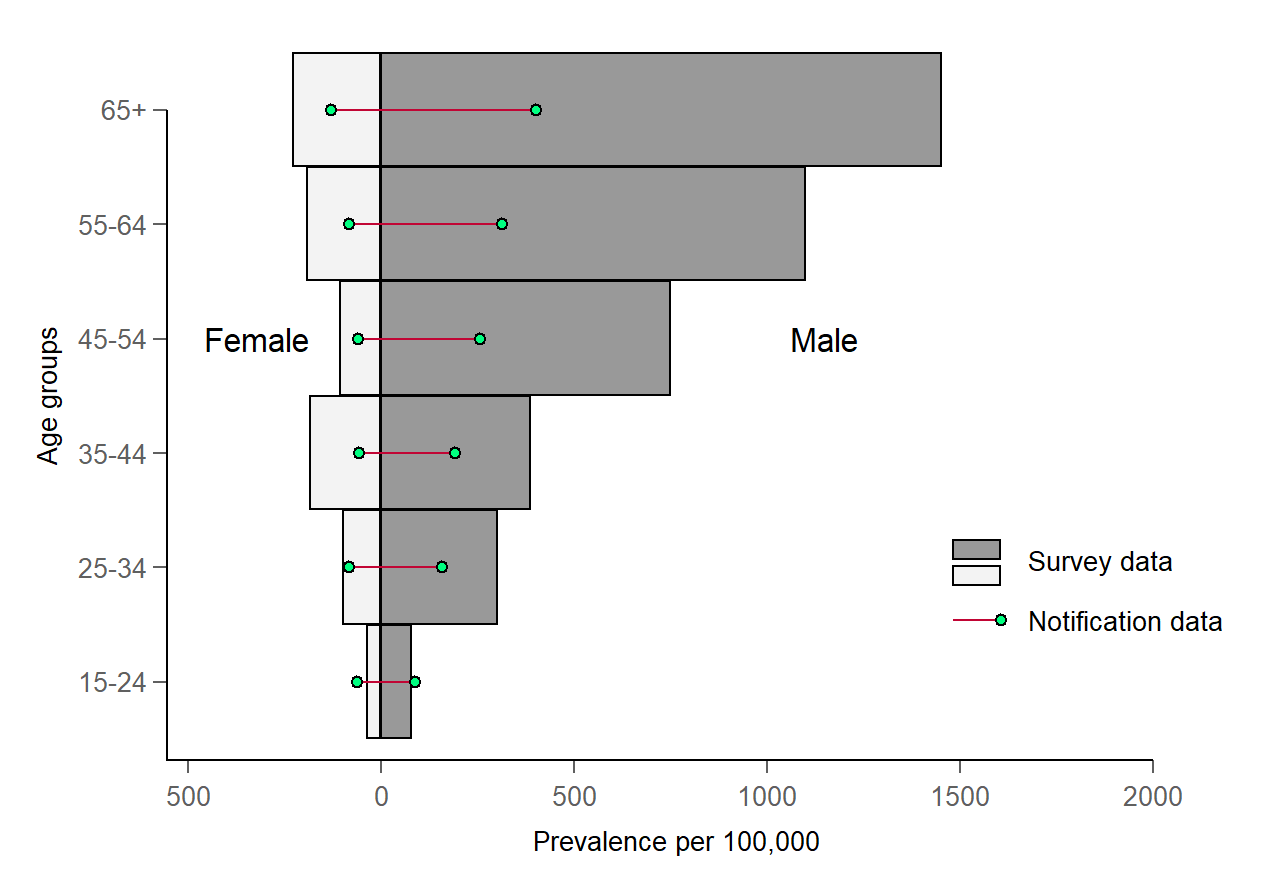

Supplement: S1 Fig — (TIF) [file pone.0232142.s003.tif]
