# Supplementary material for: The second national tuberculosis prevalence survey in Vietnam
Source: PLoS One. 2020 Apr 23;15(4):e0232142. doi: 10.1371/journal.pone.0232142 (PMC7179905; doi:10.1371/journal.pone.0232142)
Supplement: S1 Text — (DOCX) [file pone.0232142.s004.docx]

Vietnamese

**Thông tin cho đối tượng điều tra và mẫu chấp thuận tham gia**

**điều tra tình hình mắc lao toàn quốc Việt Nam lần thứ 2**

*Người phụ trách điều tra*

PGS.TS. Nguyễn Viết Nhung

TS.BS. Nguyễn Bình Hòa

Chương trình Chống lao Quốc gia Việt Nam

463 Hoàng Hoa Thám Ba Đình, Hà Nội

Điện thoại: 0912008604

**Thông tin dành cho đối tượng tham gia**

Mục tiêu chung của điều tra này là đánh giá tình hình bệnh lao tại Việt Nam hiện nay nhằm cải thiện hơn nữa công tác phòng chống bệnh lao tại Việt Nam.

Tất cả người lớn ≥ 15 tuổi đủ tiêu chuẩn sẽ nhận được giấy mời trong quá trình lên danh sách để tham gia sàng lọc bằng phỏng vấn ngắn và chụp X-Quang. Nếu có nghi ngờ lao sau khi sàng lọc, anh/chị sẽ được làm xét nghiệm hai mẫu đờm để chẩn đoán lao. Một mẫu lấy ngay tại điểm điều tra để làm xét nghiệm Xpert, một mẫu sẽ lấy tại nhà để làm nuôi cấy và soi trực tiếp.

Anh/chị được mời tham gia điều tra này vì anh/chị đang sống trong khu vực mà chúng tôi đã lựa chọn ngẫu nhiên để đưa vào điều tra.

Việc tham gia nghiên cứu là hoàn toàn tự nguyện và anh/chị có thể rút khỏi nghiên cứu bất cứ lúc nào mà không cần nói lý do.

Tất cả thông tin của anh/chị sẽ được bảo mật, lưu trữ bằng phần mềm và chỉ có những cán bộ được phân quyền tham gia nghiên cứu được tiếp cận và cũng chỉ được sử dụng vào mục đích nghiên cứu.

Nếu được chẩn đoán bị lao, anh/chị sẽ được giới thiệu đến cơ sở điều trị lao phù hợp trong mạng lưới của CTCLQG để đăng kí điều trị.

**Bản chấp thuận của đối tượng điều tra**

Tôi đã được phổ biến thông tin về việc điều tra dịch tễ lao toàn quốc lần thứ 2.

Tôi đã có cơ hội đặt câu hỏi về điều tra và nhận được những câu trả lời thỏa đáng cho câu hỏi của mình.

Bây giờ tôi đã hiểu được nếu tham gia thì tôi sẽ cần phải làm gì

Tôi hiểu rằng nếu tham gia, tôi có thể rút khỏi điều tra bất cứ khi nào mà không cần lý do.

Tôi đồng ý tự nguyện tham gia điều tra này.

Tôi không đồng ý tham gia điều tra này.

Ngày ……. Tháng…….. Năm………..

(Người tham gia ký và ghi rõ họ tên)

…………………………………………

**Dành cho cán bộ điều tra ghi:**

**Mã điểm điều tra – Mã hộ gia đình – Mã cá nhân**

⬜ ⬜ - ⬜ ⬜ ⬜ - ⬜ ⬜ ⬜ ⬜

English

**Information and consent form for participants of the 2nd national TB prevalence survey in Viet Nam**

*Persons in charge of the survey*

Associate Professor Nguyễn Viết Nhung, MD, PhD

Doctor Nguyễn Bình Hòa, MD, PhD

The Vietnam National Tuberculosis Program

463 Hoàng Hoa Thám Street, Ba Đình, Hà Nội

Tel: 0912008604

**Information for participants**

The overall objective of this survey is to evaluation the current situation of tuberculosis (TB) in Viet Nam to further improve TB control in our country.

All eligible adults ≥ 15 years of age have received invitations during the door-to-door census to paticipate in the screening procedures by an interview and chest X-ray. If you are screened positive for TB, you will be tested for TB with two sputum samples. One spot sample will be taken at the survey site for Xpert testing, the other sample will be taken at home in the next morning for direct smear microscopy and culture.

You are invited to participate in this survey because you live in an area that we selected randomly as a cluster in the survey.

Your participation in the survey is completely voluntary and you can withdraw from the survey at any time without saying the reason.

All of your information will be kept confidently, stored electronically, only assessable for authorized staffs and will only be used for research purposes.

If diagnosed with TB, you will be referred to a suitable TB treatment facility within the NTP network to register for treatment.

**Consent form for participants**

I have been informed about the 2^nd^ national TB prevalence survey.

I had the opportunity to ask questions about this survey and received satisfactory answers to my questions.

I understand what to do if I participate in the survey.

I understand that if I participate, I can withdraw from this survey at any time without any reason.

I agree voluntarily to participate in this survey.

I don’t agree to participate in this survey

Date ……. Month…….. Year………..

(Signature and full name of the participant)

…………………………………………

**For survey staff:**

**Cluster ID – Household ID – Individual ID**

⬜ ⬜ - ⬜ ⬜ ⬜ - ⬜ ⬜ ⬜ ⬜
