# Supplementary material for: The second national tuberculosis prevalence survey in Vietnam
Source: PLoS One. 2020 Apr 23;15(4):e0232142. doi: 10.1371/journal.pone.0232142 (PMC7179905; doi:10.1371/journal.pone.0232142)
Supplement: S2 Text — (DOCX) [file pone.0232142.s005.docx]

**Introduction**

In the 2^nd^ national tuberculosis prevalence survey in Vietnam (TBPS), we used two questionnaires, a screening questionnaire to screen all participants for TB symptom (cough for more than 2 weeks) and TB treatment history; and an in-depth questionnaire for the screened positive participants to assess their symptoms and access to care. We tested our survey instruments, including these questionnaires, in the pilot study conducted in two separated clusters in Nam Dinh (rural area) and Da Nang (urban area). The questionnaires are presented below, with the original Vietnamese version and the translated English version.

## BỘ CÂU HỎI PHỎNG VẤN SÀNG LỌC

**BARCODE**

MÃ SỐ CÁ NHÂN □□-□□□□

HỌ VÀ TÊN:

NĂM SINH: GIỚI:

| Q# | Câu hỏi | Trả lời |
| --- | --- | --- |
| S0 | Thông tin hiển thị trên màn hình máy tính/máy tính bảng trùng với thông tin trên Giấy mời? | Có 🡪 S1 |
|  |  | Không |
| S1 | Hiện nay anh (chị) có đang điều trị bệnh lao không? | Có (Nữ 🡪 S7, Nam 🡪 S8) |
|  |  | Không |
|  |  | Không biết |
| S2 | Anh/Chị đã từng điều trị Lao bao giờ chưa? | Có |
|  |  | Không 🡪 S4 |
|  |  | Không biết 🡪 S4 |
| S3 | Thời gian từ khi kết thúc điều trị đến nay là dưới 2 năm? | Có |
|  |  | Không |
|  |  | Không biết |
| S4 | Hiện tại, Anh/Chị có đang bị ho không? | Có |
|  |  | Không (Nữ 🡪 S7, Nam 🡪 S8) |
| S5 | Thời gian ho có trên 2 tuần không? | Có |
|  |  | Không (Nữ 🡪 S7, Nam 🡪 S8) |
|  |  | Không biết (Nữ 🡪 S7, Nam 🡪 S8) |
| S6 | Ho có khạc đờm không? *(cán bộ thực địa giải thích)* | Có |
|  |  | Không |
| S7 | Câu hỏi dành riêng cho nữ giới dưới 55 tuổi:  Chị hiện tại đang có bầu? | Có |
|  |  | Không |
| S8 | Anh/Chị có đồng ý chụp phim X-Quang để kiểm tra sức khỏe? | Có |
|  |  | Không |
| S9 | Lý do từ chối chụp phim X-Quang | Liên quan đến sức khỏe / trạng thái bệnh tật |
|  |  | Lý do khác |

Những trường hợp nghi ngờ mắc lao: Trả lời “Có” ở ít nhất một trong các câu S1, S3, S5; hoặc trả lời “Có” ở câu S7 nếu người tham gia có ho ở câu S4.

**SCREENING QUESTIONNAIRE**

**BARCODE**

PIN □□-□□□□

FULL NAME:

YEAR OF BIRTH: GENDER:

| Q# | Question text | Answers |
| --- | --- | --- |
| S0 | Is the personal data shown on screen identical to the information on the participant’s invitation? | Yes 🡪 S1 |
|  |  | No |
| S1 | Are you on TB treatment now? | Yes |
|  |  | No |
|  |  | Don’t know |
| S2 | Have you been on TB treatment before? | Yes |
|  |  | No 🡪 S4 |
|  |  | Don’t know 🡪 S4 |
| S3 | Was that treatment session ended less than 2 years ago? | Yes |
|  |  | No |
|  |  | Don’t know |
| S4 | Do you have a cough now? | Yes |
|  |  | No 🡪 (S7 for females, S8 for males) |
| S5 | Have you had this cough for 2 weeks or longer? | Yes |
|  |  | No 🡪 (S7 for females, S8 for males) |
|  |  | Don’t know 🡪 (S7 for females, S8 for males) |
| S6 | Do you produce sputum *(field worker explain details)* | Yes |
|  |  | No |
| S7 | For female participants ≤55 years of age only: Are you pregnant at the moment? | Yes |
|  |  | No |
| S8 | Do you agree to undergo chest X-ray examination? | Yes |
|  |  | No |
| S9 | Reason for refusing chest X-ray | Medical/ health-related |
|  |  | Other reason |

Screened positive: Answering "Yes" in at least one of these questions: S1, S3, S5; or answering “Yes” in question S7 if the objective confirm having cough in question S4

**BARCODE**

## PHỎNG VẤN SÂU (DÀNH CHO CÁC TRƯỜNG HỢP NGHI MẮC LAO)

MÃ SỐ CÁ NHÂN □□-□□□□

HỌ VÀ TÊN

NĂM SINH: GIỚI:

| Q# | Câu hỏi | Trả lời | |
| --- | --- | --- | --- |
| D0 | Thông tin hiển thị trên màn hình máy tính/máy tính bảng trùng với thông tin trên Giấy mời? | Có | |
|  |  | Không | |
| DD |  | | |
| DI |  |  |  |
| D1 | Trình độ văn hóa cao nhất của Anh/Chị? | Không đi học | |
|  |  | Tiểu học | |
|  |  | THCS | |
|  |  | PTTH | |
|  |  | Cao đẳng hoặc Đại học | |
|  |  | Sau đại học | |
| D1A | Tình trạng hôn nhân | Độc thân  Đã lập gia đình  Ly hôn/ly thân  Góa | |
| D2 | Anh/Chị thuộc dân tộc nào? | Kinh 🡪 D3 | |
|  |  | Khác | |
| D2b | Cụ thể dân tộc khác là dân tộc gì ? |  | |
| D3 | Anh/Chị đã từng được chẩn đoán bị tiểu đường bởi nhân viên y tế bao giờ chưa ? | Có | |
|  |  | Không | |
|  |  | Không biết | |
| D4 | Anh chị có hút thuốc lá, thuốc lào không? | Chưa từng hút thuốc 🡪D6 | |
|  |  | Đã từng hút thuốc nay đã bỏ 🡪D5 | |
|  |  | Đang hút thuốc 🡪D5 | |
| D5 | Anh chị hút trung bình bao nhiêu điếu một ngày?  *(1 điếu thuốc lào ~ 1 điếu thuốc lá)* | …………… điếu | |
|  |  |  |  |
| D5b | Anh chị hút thuốc được bao nhiêu năm? | …………… năm | |
| D6 | Trong tháng vừa qua, Anh/Chị có bị ho không? | Có | …. Tuần |
|  |  | Không 🡪D9 | |
| D7 | Trong tháng vừa qua, Anh/Chị có khạc đờm không? | Có | …. Tuần |
|  |  | Không | |
| D8 | Trong tháng vừa qua, Anh/Chị có bị ho máu không? | Có | …. Tuần |
|  |  | Không | |
| D9 | Trong tháng vừa qua, Anh/Chị có bị đau ngực không? | Có | …. Tuần |
|  |  | Không | |
| D10 | Trong tháng vừa qua, Anh/Chị có bị sốt không? | Có | …. Tuần |
|  |  | Không | |
| D11 | Trong tháng vừa qua, Anh/Chị có bị ra mồ hôi trộm về đêm không? | Có | …. Tuần |
|  |  | Không | |
| D12 | Trong tháng vừa qua, Anh/Chị có cảm thấy rất mệt mỏi không? | Có | …. Tuần |
|  |  | Không | |
| D13 | Trong tháng vừa qua, Anh/Chị có bị sụt cân không? | Có | …. Tuần |
|  |  | Không | |
|  | Nếu câu trả lời từ D6 đến D13 là Không 🡪D21 | | |
| D14 | Anh/Chị có tìm cách chữa trị triệu chứng kể trên không không? | Có | |
|  |  | Không 🡪D17 | |
| D15 | Bao nhiêu tuần trước đây Anh/Chị bắt đầu tìm kiếm các dịch vụ chữa trị triệu chứng này? | Điền số tuần ......  *Nếu người được phỏng vấn trả lời bằng số tháng thì cán bộ phỏng vấn chuyển thành số tuần (lấy trung bình 1 tháng = 4 tuần)* | |
| T 1 | Lưu ý cho cán bộ phỏng vấn:  Đánh số theo thứ tự 1 đến tối đa là 7 cho các cơ sở y tế mà người được phỏng vấn đã lần lượt đến khám: 1 là cơ sở y tế đầu tiên, 2 là cơ sơ y tế thứ hai, ... Nếu người được phỏng vấn đến một cơ sở y tế nhiều lần, thì chỉ ghi lại theo lần đến khám đầu tiên Nếu không đến bất cứ cơ sở y tế nào thì ghi là '0' | | |
| D16 | Anh/Chị từng đi khám hoặc tư vấn ở những cơ sở y tế/nhà thuốc nào? (Nơi nào anh chị tới đầu tiên là số 1, nơi tiếp theo là số 2....) | ….. Trạm y tế xã | |
|  |  | ….. Bệnh viện quận/huyện | |
|  |  | ….. Bệnh viện/Trung tâm chống Lao | |
|  |  | ….. Bệnh viện tỉnh | |
|  |  | .... Bệnh viện trung ương | |
|  |  | ….. Nhà thuốc | |
|  |  | ….. Phòng khám tư | |
|  |  | ….. Khác 🡪D16b | |
| D16b | Cung cấp thông tin chi tiết về loại hình dịch vụ y tế: |  | |
| D17 | Do các triệu chứng trên, Anh/Chị đã được chụp phim X-Quang chưa ? | Có | |
|  |  | Không 🡪D19 | |
| D18 | Phim X-Quang được chụp tại cơ sở y tế công hay cơ sở y tế tư? | Cơ sở y tế công | |
|  |  | Cơ sở y tế tư | |
| D19 | Do các triệu chứng trên, Anh/Chị đã được làm xét nghiệm đờm chưa ? | Có | |
|  |  | Không 🡪D21 | |
| D20 | Xét nghiệm đờm được thực hiện tại cơ sở y tế công hay cơ sở y tế tư? | Cơ sở y tế công | |
|  |  | Cơ sở y tế tư | |
| D21 | Anh/Chị đã từng điều trị bệnh Lao? | Có 🡪D22 | |
|  |  | Không | |
| D21b | Anh/Chị hiện đang có các triệu chứng giống như các triệu chứng Anh/Chị đã khai báo ở phần D6-D13? | Có  Không | |
|  | Trước đây, Anh/Chị đã có triệu chứng của bệnh phổi không? | Có  Không | |
|  | Anh/Chị đã từng đi chụp phim X-Quang phổi trước đây? | Có  Không | |
|  | Anh/Chị đã từng đi làm xét nghiệm đờm trước đây? | Có  Không | |
|  | Anh/Chị đã uống thuốc điều trị lao kéo dài trên một tháng không? | Có  Không | |
|  | Anh/Chị đã từng tiêm thuốc kéo dài trên một tháng? | Có  Không | |
| D21c | Sau những câu hỏi trên, anh chị có nhớ lần điều trị lao trước đây? | Có  Không 🡪D23 | |
| D22 | Anh/Chị được điều trị lao khi nào? | Từ tháng ……, năm ……….  đến tháng ……, năm ………… | |
| D22b | Anh/Chị được điều trị lao ở đâu (chọn tất cả các cơ sở y tế anh chị được điều trị lao)? | Trạm y tế xã  Bệnh viện/TTYT tuyến quận/huyện  Bệnh viện/trung tâm chống lao  Bệnh viện tỉnh  Bệnh viện tuyến trung ương  Nhà thuốc  Phòng khám tư  Khác:…………………. | |
| D23 | Anh/Chị mất bao nhiêu thời gian để đi đến bệnh viện gần nhất (với phương tiện giao thông Anh/Chị đang sử dụng)? | …. Giờ ……… phút  Bằng phương tiện…………  Ô tô  Xe máy/xuồng máy  Xe đạp  Đi bộ  Khác …….. | |
| D24 | Anh/Chị mất bao nhiêu thời gian để đi đến trạm y tế phường/xã gần nhất (với phương tiện giao thông Anh/Chị đang sử dụng)? | …. Giờ ……… phút  Bằng phương tiện…………  Ô tô  Xe máy/xuồng máy  Xe đạp  Đi bộ  Khác …….. | |
| *T2* | Cám ơn Anh/Chị đã trả lời các câu hỏi phỏng vấn. Anh/Chị có câu hỏi nào dành cho chúng tôi hay không? | | |

**BARCODE**

## INDEPTH INTERVIEW (SCREENED POSITIVE ONLY)

PERSONAL ID □□-□□□□

FULL NAME

YEAR OF BIRTH GENDER

| Q# | Question | Answers options | |
| --- | --- | --- | --- |
| D0 | Is the personal information identical on the computer/tablet and the survey card? | Yes  No | |
| DD |  | | |
| DI |  |  |  |
| D1 | What was is the highest education that you have completed? | No schooling | |
|  |  | Primary school | |
|  |  | Secondary school | |
|  |  | High school | |
|  |  | College or university | |
|  |  | Further education after college/university | |
| D1b | Marital status: | Single  Married  Divorced / separated  Widowed | |
| D2 | Of what ethnic group do you consider yourself? | Kinh 🡪 D3 | |
|  |  | Other | |
| D2b | Please specify your ethnic group: |  | |
| D3 | Have you ever been diagnosed with diabetes by a health worker? | Yes | |
|  |  | No | |
|  |  | Don’t know | |
| D4 | Do you smoke? | Never smoke 🡪 D6 | |
|  |  | Used to smoke 🡪 D5 | |
|  |  | Currently smoke 🡪 D5 | |
| D5 | On average, how many cigarettes do you smoke a day?  *(1 pipe-cigarette shot ~ 1 cigarette)* | ……… cigarettes | |
|  |  |  |  |
| D5b | How many year have you smoked? | ……… years | |
| D6 | In the past month, did you have cough? | Yes | …… weeks |
|  |  | No 🡪 D9 | |
| D7 | In the past month, did you cough up sputum? | Yes | …… weeks |
|  |  | No | |
| D8 | In the past month, did you cough up blood? | Yes | …… weeks |
|  |  | No | |
| D9 | In the past month, did you have chest pains? | Yes | …… weeks |
|  |  | No | |
| D10 | In the past month, did you have fever? | Yes | …… weeks |
|  |  | No | |
| D11 | In the past month, did you have night sweats? | Yes | …… weeks |
|  |  | No | |
| D12 | In the past month, did you feel very tired? | Yes | …… weeks |
|  |  | No | |
| D13 | In the past month, did you lose any weight? | Yes | …… weeks |
|  |  | No | |
|  | If answers of D6 to D13 are “No” 🡪 D21 |  | |
| D14 | Did you seek healthcare to deal with the above health problems? | Yes  No | |
| D15 | How long ago (in weeks) did you seek healthcare to deal with the above health problems? | ...... weeks  *If participants answer in month, convert into week (on avarage, 1 month~ 4 weeks)* | |
| T 1 | Note for interviewer:  *Mark 1 to maximum 7 to represent the order in which the person visited these facilities; 1 being the first facility, 2 the second, etc.*  *If participants visited same provider more than once, write only the first visit*  *If not visited, write* ‘0’ | | |
| D16 | Did you seek treatment or advice at any of the following? (1 is the first place you came for treatment/advice, 2 is the second place, etc.) | …… Commune Health Station | |
|  |  | …… District hospital | |
|  |  | …… TB hospital/ Center | |
|  |  | …… Provincial hospital | |
|  |  | …… National hospital | |
|  |  | …… Drug store | |
|  |  | …… Private clinic | |
|  |  | …… Other 🡪 D16b | |
| D16b | Please specify the type of provider: |  | |
| D17 | Due to these symptoms, have you had chest X-ray examination? | Yes | |
|  |  | No 🡪 D19 | |
| D18 | Did you get chest X-ray at public or private facility? | Public facility | |
|  |  | Private facility | |
| D19 | Due to these symptoms, have you had sputum test? | Yes | |
|  |  | No 🡪 D22 | |
| D20 | Did you get sputum test at public or private facility? | Public facility | |
|  |  | Private facility | |
| D21 | Have you ever been treated for tuberculosis? | Yes 🡪 D22  No | |
| D21b | Have you had these same symptoms you’ve just reported in question D12-D18? | Yes  No | |
|  | Have you had other symptoms of lung disease in the past? | Yes  No | |
|  | Have you had X-ray examinations in the past? | Yes  No | |
|  | Have you had sputum examinations in the past? | Yes  No | |
|  | Have you ever taken TB drugs for more than one month? | Yes  No | |
|  | Have you ever had injections for more than one month? | Yes  No | |
| D21c | After these questions, do you now remember previous TB treatment? | Yes | |
|  |  | No 🡪 D23 | |
| D22 | When were you treated for tuberculosis? | From <mm/yyyy> to <mm/yyyy> | |
| D22b | Where were you treated for tuberculosis? (provide name and place of health facility) | □ Commune Health station  □ District hospital  □ TB hospital/ centre  □ Provincial hospital  □ National hospital  □ Drug store  □ Private clinic  Other:…………………. | |
| D23 | How long does it take you to travel to nearest hospital? (by which vehicle you typically use) | ……… minutes  By …………………  Car  Motorbike/ canoe  Bike  Walking  Other……… | |
| D24 | How long does it take you to travel to nearest community health post (by the transportation means that you would typically use) | ……… minutes  By …………………  Car  Motorbike/ canoe  Bike  Walking  Other……… | |
| *T2* | Thank you for answering all these questions. Do you have any questions? | | |
